# Susceptibility to movement-evoked pain following resistance exercise

**DOI:** 10.1371/journal.pone.0271336

**Published:** 2022-07-21

**Authors:** Einat Kodesh, Anat Sirkis-Gork, Tsipora Mankovsky-Arnold, Simone Shamay-Tsoory, Irit Weissman-Fogel

**Affiliations:** 1 Department of Physical Therapy, University of Haifa, Haifa, Israel; 2 Department of Psychology, University of Haifa, Haifa, Israel; Universiteit Antwerpen, BELGIUM

## Abstract

**Objective:**

To investigate the: (1) role of basic muscle pain sensitivity and psychological factors in the prediction of movement-evoked pain (MEP) following delayed onset muscle soreness (DOMS), and (2) association of MEP with changes in systemic muscle pain sensitivity following DOMS induction.

**Methods:**

Fifty-one participants were assigned to either eccentric resistance exercise or control groups. They completed questionnaires evaluating psychological distress and underwent muscle pain sensitivity evaluation by the pressure pain threshold (PPT) test at the exercised and remote muscles, before and 24 hours following the intervention. MEP intensity was determined in response to lifting a 3kg canister using a visual analogue scale (VAS).

**Results:**

The exercise group demonstrated MEP intensity of 5/10 on VAS and reduced PPTs at the main exercised muscle (p<0.001). A regression tree analyses revealed that the level of anxiety trait predicted a higher MEP intensity. A secondary analysis showed that 53% participants who were DOMS responders (MEP > mild intensity; ≥ 3/10 VAS) exhibited decreased PPTs in the exercised (p<0.001) and remote (p = 0.027) muscles following eccentric exercise. Characterization of DOMS responders revealed that, at baseline, they had lower PPTs in the exercised (p = 0.004) and remote (p = 0.001) muscles and reported higher psychological distress i.e., anxiety trait and depression symptoms (p<0.05), compared to non-responders. A regression analysis revealed that lower PPT or high levels of anxiety trait increased the probability to become a responder (p = 0.001).

**Conclusions:**

Susceptibility to MEP following DOMS is determined by muscle pain hypersensitivity and high levels of anxiety trait. MEP at the early stage of DOMS is linked with an increase in systemic muscle pain sensitivity suggestive of central mechanisms. This knowledge is valuable in translating science into clinical musculoskeletal pain management.

## Introduction

Delayed onset muscle soreness (DOMS) is an acute muscle pain condition that reaches its peak at 24–72 hours, usually following eccentric contractions [1–4]. In order to evaluate DOMS intensity, previous studies used pain recalled through self-report questionnaires or spontaneous pain ratings. However, an assessment of pain in general, and not specifically in relation to evoked movement, does not evaluate the main clinical characteristic of DOMS, which is commonly referred to as movement-evoked pain (MEP). Because DOMS is often used as a model of acute muscle pain in experimental pain research in which the effects of interventions on muscle pain are tested [5], it is important to use an objective test such as MEP as an outcome [6–8].

Mechanisms implicated in MEP associated with musculoskeletal pains are multifactorial including peripheral and central mechanisms [7]. Yet to the best of our knowledge, only one study investigated MEP mechanisms following DOMS induction and it focused on brain-related activity [9]. MEP in the exercised muscle following DOMS induction is a type of mechanical allodynia i.e., pain evoked in response to movement only and not under normal conditions. In fact, large diameter (myelinated) afferents [10, 11] or C-tactile (CT) fibers [12] might underlie the mechanical allodynia observed in DOMS, presumably via centrally mediated mechanisms i.e., central sensitization. This central neuroplasticity accompanies the peripheral sensitization of nociceptive neurons [13, 14]. The latter is due to inflammatory processes caused by ultrastructural muscle damage in the exercised muscles [15, 16]. In detail, biopsy analysis of eccentric strained muscle tissue shows that there is a disruption of sarcomeres which leads to protein degradation and a local inflammatory response [2, 17]. Furthermore, a higher cytokine concentration also acts to mediate inflammation and increase vascular permeability [18]. In addition, the ultrastructural muscle damage is associated with increased cytosolic calcium concentrations, which activate proteolytic enzymes and contribute further to vascular permeability [19]. The accumulation of interstitial fluid is accompanied by intramuscular edema which is responsible for nociceptor activation and pain sensation. The poor correlation observed between perceived pain intensity and the degree of change of biological markers of muscle damage [20–22] further supports the key role of central mechanisms in mechanical allodynia following DOMS induction. Furthermore, central mechanisms have been implicated in MEP in individuals with other musculoskeletal pains (e.g. knee osteoarthritis) as evident of its association with temporal summation [23, 24] and not with spontaneous pain [23]. Thus, one aim of this study was to understand whether MEP following DOMS induction is associated with potential central neuroplastic changes manifested as a reduction in systemic pain sensitivity i.e., a decrease in pressure pain thresholds (PPT) in remote areas from the exercised muscles.

In addition to the neurophysiological mechanisms, MEP also involves complex relationships and interactions with negative psychological factors (e.g., pain catastrophizing, fear-avoidance, depression) [6, 7]. These factors may contribute to inter-subject variability in the severity of DOMS. Indeed, DOMS intensity, not evaluated specifically in relation to MEP, varies from slight stiffness in the muscles that disappears rapidly with the carrying out of routine daily activities to severe debilitating pain that interferes with movement [2]. Therefore the severity of DOMS is substantially influenced by both individual differences in pain sensitivity [25, 26] and by psychological constructs [27, 28]. However, these factors have not been tested in relation to DOMS evaluated by the MEP test. Accordingly, this study focused on MEP as a procedure for DOMS assessment and aimed at investigating: 1) the predictive role of individual pain sensitivity and psychological constructs on MEP intensity, and 2) the association of DOMS-induced MEP with changes in systemic pain sensitivity.

## Material and methods

### Study population

Healthy sedentary individuals aged 18–40 years were recruited. Participants were excluded from the study if they 1) engaged in exercise or sporting activities more than once a week within the previous six months; 2) reported physiological, psychiatric, cognitive or neurological disorders, or any acute or chronic pain condition; 3) used medications on a regular basis; or 4) were pregnant. Participants were asked to avoid analgesic medication at least 24 hours prior to session 1 through to the end of session 2.

### Study procedure

The study was approved by the Institutional Research Board of the University of Haifa (ethics approval # 070/16) and took place at the university’s Physical Therapy Laboratories. Potential study participants were screened via a telephone interview and then those that met the inclusion criteria were asked to attend two experimental sessions. In the first session participants gave their signed informed consent and were assigned to either the eccentric resistance exercise group or the control group based on stratified randomization using Microsoft Excel software and controlling for age and sex. Demographic and anthropometric information was collected, and participants underwent familiarization with the PPT test. Thereafter, they were asked to lift a 3 kg canister for the MEP evaluation and to rate the level of pain using the 10 cm visual analogue scale (VAS) scale in the McGill Pain Questionnaire-short form (SF-MPQ) [29]. Following this, all participants completed psychological questionnaires and the PPT tests. Finally, those allocated to the exercise group only underwent the eccentric exercise protocol for DOMS induction.

**Fig 1:**
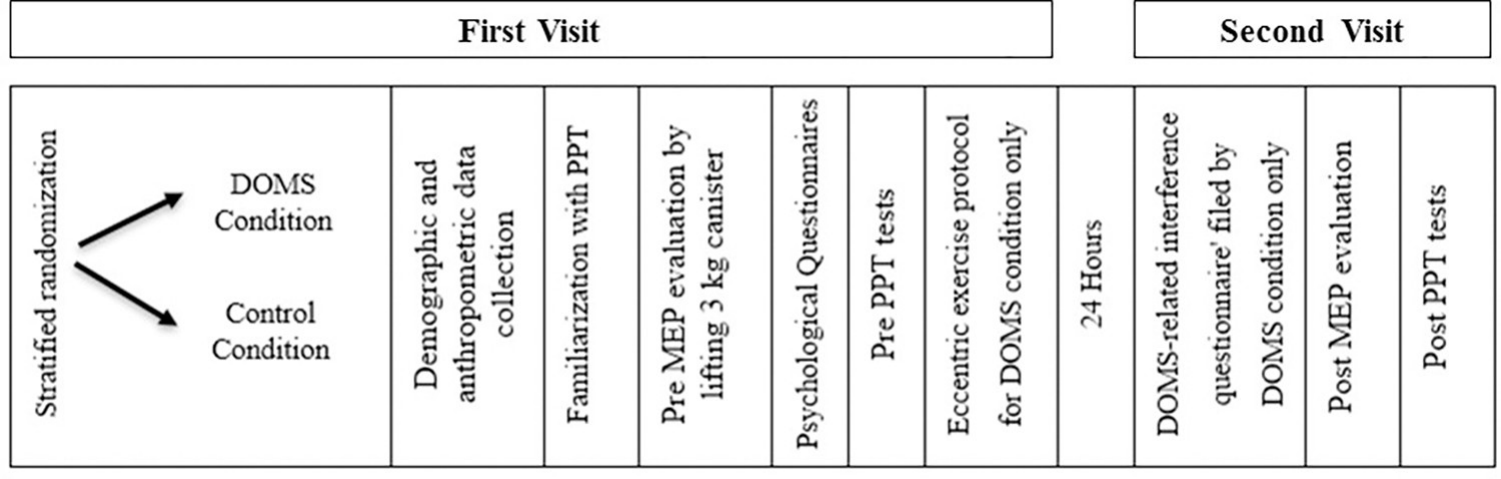
Flow chart of study procedure.

All participants returned to the laboratory 24 hours after the first session. The exercise group who had undergone the DOMS protocol filled out the DOMS-related interference questionnaire and then again underwent the MEP evaluation. Thereafter, all participants, regardless of their group assignment, completed the PPT tests in the same order as in the first session (Fig 1).

### Eccentric exercise protocol

Participants underwent a brief familiarization of the eccentric exercise protocol where they were shown the correct way to carry out the exercises. Each participant then carried out the exercise protocol. Dumbbell weight was adjusted to each participant and for each exercise. Three unilateral [30, 31] exercises for right upper extremity was performed in a standing position: 1) biceps curl to target the biceps muscle; 2) shoulder flexion to target the anterior deltoid muscle; and 3) chest press to target the pectoralis major.

To determine the appropriate weight load for each participant, a 5-repetition maximum (5RM; i.e. the maximum weight lifted with 5 concentric contractions) strength test was conducted. Participants were asked to estimate their 5RM and this weight then served as the starting weight. The load was then adjusted until the participant could not complete more than 5 repetitions; this load then served as the 5RM load. This 5RM strength test was conducted for each of the three exercises. The trials carried out until finding each participant’s 5RM load were separated by 3-minute rest periods. Participants were asked to perform 10 sets of 5RM for each exercise [30] The exercise was performed while the investigator assisted the participant in lifting the load in the concentric phase. In order to emphasize eccentric contractions, participants were asked to control the eccentric phase repetition for approximately 10 seconds while attempting to maintain a constant velocity throughout the movement. One-minute rest breaks were taken between sets, and two-minute rest breaks were taken between exercises [32]. After each set, participants rated their perceived exertion on a scale between 6 "no effort " to 20 "maximal effort" [33].

DOMS intensity was determined in response to lifting a 3 kg canister (i.e. the MEP test) filled with sand [32] using the 10 cm VAS scale from the SF-MPQ [29]. This measure represents the pain evoked by a physical task performed at one point in time, 24 hours after conducting the eccentric exercise protocol. In detail, the canister was positioned on a table that its height was adjusted so that the handle of the canister was at standing elbow height. The participant stood erect with his or her elbow bent at 90 degrees and he was asked to lift the canister with arm fully extended.

### DOMS intensity measures

#### DOMS-related interference questionnaire

We used a modified version of the questionnaire used by Trost and colleagues [34] which refers to back pain. DOMS-related interference was assessed by asking the participant the following question: “Did the pain in your upper extremity prevent you from performing your usual activities in the past day?”. The participant was asked to provide their responses on a 1–4 Likert scale.

#### McGill Pain Questionnaire-short form (SF-MPQ)

MEP intensity was quantified using the SF-MPQ in response to lift a 3 kg load to the maximum reach prior (1^st^ session) and 24 hours following (2^nd^ session) DOMS protocol implementation. In the SF-MPQ three pain assessment tools are included: the pain rating index (PRI), the present pain intensity index (PPI) and a VAS [29]. The PRI contains a total of 15 descriptors, which are rated on a 4-point Likert scale for pain intensity with answers ranging from 0 (‘none’) to 3 (‘severe’). For each session, a pain score was derived by calculating the sum of the intensity rank values for all 15 descriptors. For the PPI, participants were asked to indicate the intensity of pain on a 6-point Likert scale, with answers ranging from 0 (‘no pain’) to 5 (‘excruciating pain’). The VAS was measured on a scale ranging from 0 to 10. The VAS scores were used for quantification of the MEP intensity.

#### Muscle pain sensitivity measurement

We used the PPT test for evaluation of muscle pain sensitivity [35]. All participants completed a training session. This included familiarization with the PPT test in which three repetitions of the PPT test were applied to the exercised hand thenar eminence. The study PPTs were tested at the exercised muscles (i.e., right deltoid and biceps) and in a remote muscle (i.e., left forearm flexors) using a pressure algometer (Somedic Algometer, Sweden). At each of the three selected sites [right anterior deltoid (PPTd), right biceps (PPTb) and left flexor (PPTf)], 3 pressure stimuli were administered, with an increasing rate of 30 kPa/sec and an inter-stimulus interval of 30 sec. The participant was asked to push a button to indicate the point at which the pressure stimulus was perceived as minimally painful. PPT was computed as the mean of the 3 measures.

### Pain-related psychological questionnaires

#### Pain Catastrophizing Scale (PCS)

The 13 item PCS [36] was used in order to assess catastrophic thinking related to past painful experiences. A 5-point Likert scale was used to rate their responses where 0 = ‘not at all’ to 4 = ‘all the time’. The PCS total score is computed by summing responses to all 13 items and may range from 0–52. The PCS has been shown to have high internal consistency (coefficient alpha = .87) and the Hebrew version has demonstrated good reliability (coefficient alpha = .86) [37].

#### Spielberger’s State-Trait Anxiety Inventory (STAI)

Anxiety level was assessed by Spielberger’s STAI using the validated Hebrew version [38]. Part I of the questionnaire assesses the level of state anxiety and Part II assesses the level of trait anxiety. Each part includes 20 statements which describe various emotional states. Participants were asked to rate each item based on a 4-point Likert scale (1–4), ranging from 1-‘not at all’ to 4-"very much so’ for the STAI state scale and from ‘almost never’ to ‘almost always’ for the STAI trait scale. Scores range from 20 to 80, with higher scores suggesting greater levels of anxiety. The STAI has good internal consistency (coefficient alpha >.89), and the STAI trait scale has evidenced good test–retest reliability (average r = .88) at multiple time intervals [39].

#### Beck Depression Inventory (BDI)

The BDI is a 21-item self-report measure of the severity of depressive symptoms [40]. Participants rated the severity of each symptom on a 4-point scale ranging from 0 to 3. A total score (range 0–63) was computed by summing individual items’ scores and was interpreted as follows: normal (0–9), light depression (10–18), mild depression (19–26). A total score above 26 is considered to be indicative of severe depression. The BDI has demonstrated good reliability and validity (coefficient alpha = .81) [41].

#### Perceived Stress Scale (PSS)

The PSS Hebrew version [42] is comprised of a 10-item self-report measure of the degree to which common life situations are evaluated as stressful. Participants were asked to respond to each situation (relating to the last month) on a 5-point Likert scale, with answers ranging from 0 (‘never’) to 4 (‘very often’). Higher scores on this measure indicate greater levels of perceived stress. The PSS has been shown to have high internal consistency (coefficient alpha = .84-.86) and a good test–retest reliability (average r = .85) [42].

#### Fear of Pain Questionnaire (FPQ) Short Form

The FPQ [43] was used to measure pain-related fears. The FPQ-SF is a 20-item self-report inventory designed to assess fear of specific pain causing stimuli. Research supports the internal consistency (coefficient alpha = .91) and construct validity of the FPQ-SF [43].

### Statistical analysis

A priori power analysis using the G*power computer program [44] indicated that a total sample size of 34 people would be needed to detect medium effects of η2p = 0.06 with 80% power and alpha at .05 using an ANOVA repeated measure, within between interactions.

Data are expressed as mean ± SD or median and interquartile range, as appropriate according to the normality distribution assessed by the Shapiro-Wilks test.

A two way 2 (group: exercise; control) * 2 (time: 1^st^ session; 2^nd^ session) mixed-model repeated-measures analysis of variance (ANOVA) was run to examine the effect of group and time on changes in PPT, and the interaction between them. This was done separately for each PPT measure (PPTd, PPTb, and PPTf). A simple mean analysis was conducted to further explore differences between the groups and we used the post hoc Bonferroni Procedure for multiple comparisons (4 comparisons).

For a secondary exploratory analysis the participants in the exercise group were divided into responders and non-responders to DOMS-related MEP based on Mankovsky-Arnold et al.’s study [32]. We defined responders if MEP ratings at the beginning of sessions 2 was VAS≥3 (above mild pain). Those with VAS of 2 or lower were defined as "non-responders". In order to characterize responders vs. non-responders, we performed univariate analysis using unpaired t-tests or Mann-Whitney tests as appropriate for baseline anthropometric, demographic, pain-related psychological factors, and PPTs. To investigate changes in pain sensitivity following DOMS induction in relation to responsivity to MEP, we applied two-way mixed-design ANOVA; time (1^st^ session; 2^nd^ session) defined as the within-subjects factor, group (responders; non-responders; controls) as between-subjects factor. We tested the main effect of time, group, and the interaction between time and group. This was done separately for each PPT measure (PPTd, PPTb, and PPTf). A simple mean analysis was conducted to further explore differences between groups and we used the post hoc Bonferroni Procedure for multiple comparisons (6 comparisons).

We conducted decision and regression tree analyses with the R party package, using random forest variable selection [45] and Monte Carlo simulation for multiple-testing adjustment. These were carried out in order to predict the probability of MEP occurrence and its intensity and to explore interactions between potential predictors. The potential predictors were variables with associated p values of < 0.05 on univariate analysis including the PPTf, PPTb, PPTd anxiety trait, BDI, PSS, and sex. In order to control for dumbbell weight used during DOMS induction, individual muscle load for Biceps curl and Deltoid were added as a covariate.

Statistical analyses were performed using SAS version 9.4 for Windows and the R Foundation for Statistical Computing version 3.6.1. Statistical significance was taken as p <0.05.

## Results

### Participants

Fifty-one (23 males) healthy subjects were recruited to the study, aged 25.9±4.9 years. The exercise group included 32 participants (14 males). The control group included 19 participants (9 males). No significant differences were found for age (t_42.2_ = -0.37, p = 0.730), weight (t_28.6_ = 0.51, p = 0.609), or height (t_32.9_ = -0.75, p = 0.434) between the groups. Psychological measures were not significantly different between groups (Table 1). Also, no group differences were found at baseline (1st session, before DOMS induction) for PPTs tested at the forearm flexors (t_25.3_ = -0.68, p = 0.502); Biceps (t_20.2_ = -1.09, p = 0.290) and Deltoid (t_19.5_ = -1.07, p = 0.298) (PPTs values are presented in Table 2).

**Table 1:** Scores of psychological factors in the exercise and control group.

Psychological factors (questionnaires)	Exercise Median (Q1, Q3) n = 32	Control Median (Q1, Q3) n = 19	p-value
**Pain catastrophizing** (PCS)	22.5 (14.0, 33.5)	26.0 (17.0–35.0)	0.72
**Trait anxiety** (STAI)	43.0 (40.5, 46)	44.5 (42.0–47.0)	0.48
**State anxiety** (STAI)	44 (39.0–46.5)	43 (40.0–45.0)	0.64
**Depression** (BDI)	7.5 (4.5,12.5)	7.5 (3.0,13.0)	0.83
**Fear of pain** (FPQ)	76.5 (67.0, 91.5)	74.00 (67.0, 82.0)	0.45
**Perceived Stress Scale** (PSS)	21.0 (19.0, 24.0)	21.0 (16.0, 23.0)	0.48

PCS, pain catastrophizing scale; STAI, Spielberger’s State-Trait Anxiety Inventory; BDI, Beck Depression Questionnaire; FPQ, Fear of Pain Questionnaire; PSS, Perceived Stress Scale (PSS).

**Table 2:** PPT’s values at baseline (1^st^ session) and following 24 hours (2^nd^ session).

QST parameters	Exercise n = 32			Controls n = 19		
	Session 1	Session 2	% Change	Session 1	Session 2	% Change
	Mean (SD)	Mean (SD)	Mean (SD)	Mean (SD)	Mean (SD)	Mean (SD)
**PPTf kPa**	426.7 (110.6)	357.01 (124.6)	15.00 (20.6)	457.5 (191.2)	435.2 (237.9)	5.0 (22.3)
**PPTb kPa**	305.9 (86.5)	237.8 (90.3)	16.05 (35.1)	355.9 (178.6)	326.7 (169.07)	5.0 (26.5)
**PPTd kPa**	337.9 (102.25)	264.7 (121.2)	21.5 (27.2)	400.7 (229.8)	403.8 (237.6)	3.4 (32.09)

PPTf, pressure pain threshold left forearm flexor; PPTb, pressure pain threshold right biceps; PPTd, pressure pain threshold right deltoid.

### Change in pain sensitivity following eccentric exercise

After 10 sets of 5RM participants could no longer control the descent of the weight and their rate of perceived exertion in each of the exercises reached the following averages: 19.4±0.8 out of 20 for the Biceps, 19.8±0.7 for the Deltoid, and 19.7±0.9 for the Pectoralis, evident of peak activity in the involved muscles.

The DOMS intensity measures 24 hours following DOMS induction in the exercise group were: median (Q1, Q3) for the PRI 4.5 (4.3, 10.3), the PPI 2.0 (1.4, 2.4), the MEP 3.0 (2.0, 3.8) and for DOMS interference 2.5 (2.3, 3.4). The PPTs values for the exercise and the control group at 1^st^ and the 2^nd^ session (24 hours following the intervention) are shown in Table 2. Main effect of time was statistically significant for the PPT tested at the Biceps (F _(1, 37.3)_ = 13.39 p = 0.0024, ɳ^2^_p_ = 0.012), and Forearm Flexors (F_(1,32)_ = 8.41 p = 0.018, ɳ^2^_p_ = 0.077) yet not for the Deltoid (F_(1,31.8)_ = 5.39, p = 0.081, ɳ^2^_p_ = 0.050). No main effect of group was found. Furthermore, no group and interaction effects were found for the PPTb and PPTf. Yet, a group by time interaction was found for PPT at the Deltoid (F_(1,31.8)_ = 6.38 p = 0.048, ɳ^2^_p_ = 0.060). A simple mean analysis revealed that only the exercise group demonstrated a significant increase in pain sensitivity following eccentric exercise as expressed by reduction in PPT (exercise diff = 73 t_30_ = 4.20, p<0.001, 95% CI(37.57, 108.82), *d*_*z*_ = 0.65; controls diff = -3.0 t_16_ = -0.13, p = 0.90, 95% CI(-55.35,49.16), *d*_*z*_ = 0.013).

### Prediction of DOMS-related MEP intensity

**Fig 2:**
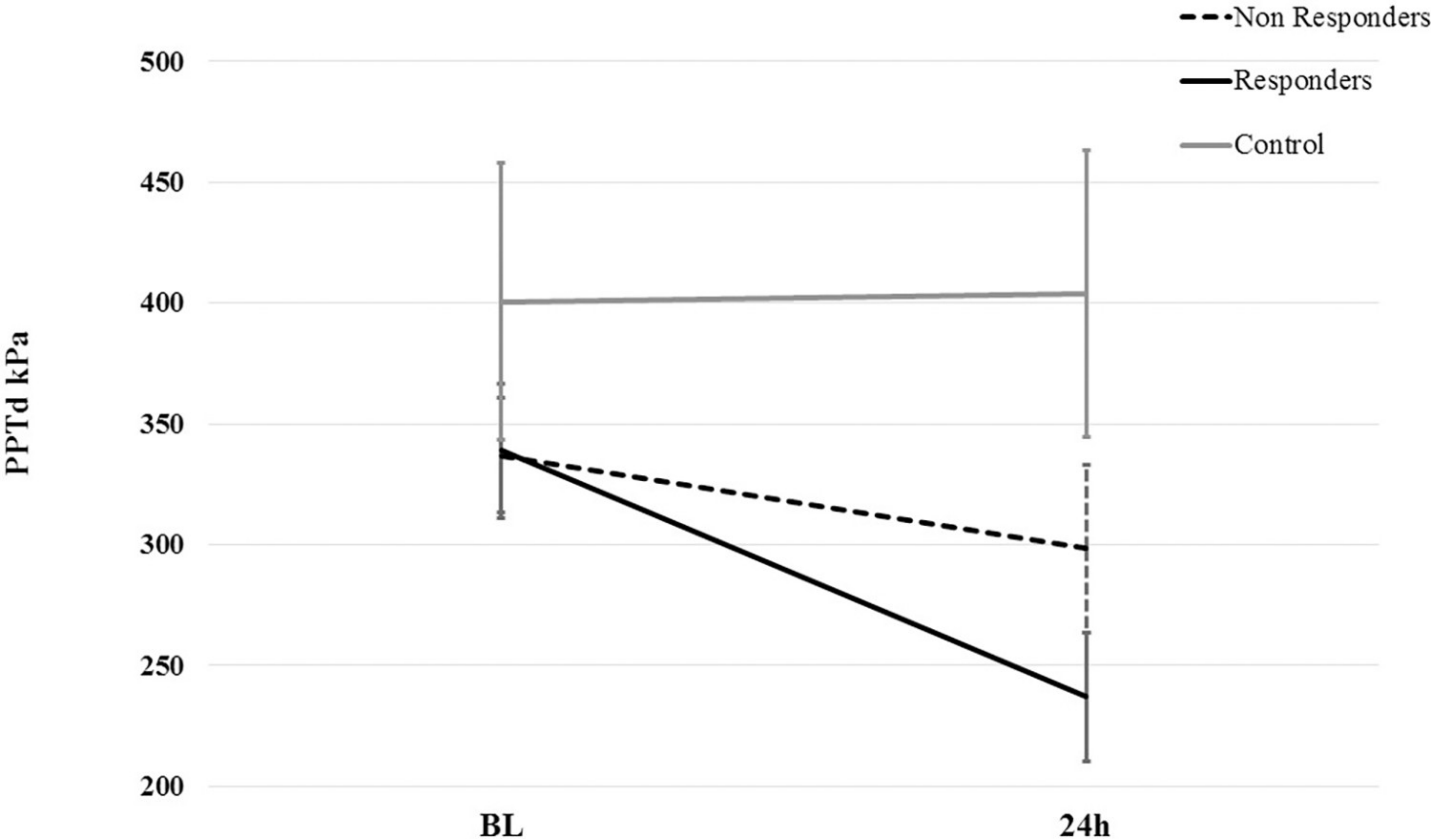
The tree regression analysis to predict DOMS intensity. A regression tree analysis was set up to predict DOMS pain intensity by anxiety score below or above 46 and sex. The numbers in the circles indicate the level of statistical significance (P); and the numbers in the rectangular boxes reflect the total number of cases for that outcome (n) and the mean of pain intensity (y).

The tree regression analysis to predict DOMS intensity (Fig 2) revealed a significant split in the anxiety trait scores. When the trait anxiety scores were >46 the mean pain intensity was 5.4 VAS (out of 10) regardless of sex (this was true for 7 participants). However, female participants with anxiety trait scores ≤46 showed a higher mean pain intensity than males with the same anxiety levels (females = 3.38; males = 1.25 VAS).

### Secondary exploratory analysis (responder vs. non-responders)

We found that out of the 32 participants in the exercise group, 17 (53%) showed a DOMS above mild intensity (i.e. VAS≥3) in response to MEP and defined as responders (as explained on the Methods section). There were more females than males who were DOMS responders (14 [72%] females vs. 4 [29%] males; p = 0.014).

Table 3 summarizes the of DOMS intensities 24 hours after the DOMS administration. Significant differences were found between DOMS responders and non-responders in PRI (U _(17.88)_ = -3.88, p<0.001), PPI (U _(24.44)_ = -2.56, p = 0.002), MEP (U _(26.1)_ = -7.24, p<0.0001), and DOMS interference (U _(26.1)_ = -7.24, p<0.001).

**Table 3:** DOMS intensity in responders and non-responders.

	Responders (n = 17) Median (Q1, Q3)	Non-responders (n = 15) Median (Q1, Q3)
**MEP** *	5 (3, 6)	1 (0, 2)
**PRI** *	10 (3, 15)	3 (1, 4)
**PPI** *	3 (2, 3)	1 (0, 2)

MEP, movement evoked pain scale; PRI, Pain Rating Index; PPI, Present Pain Intensity Index

*denotes p<0.05.

**Fig 3:**
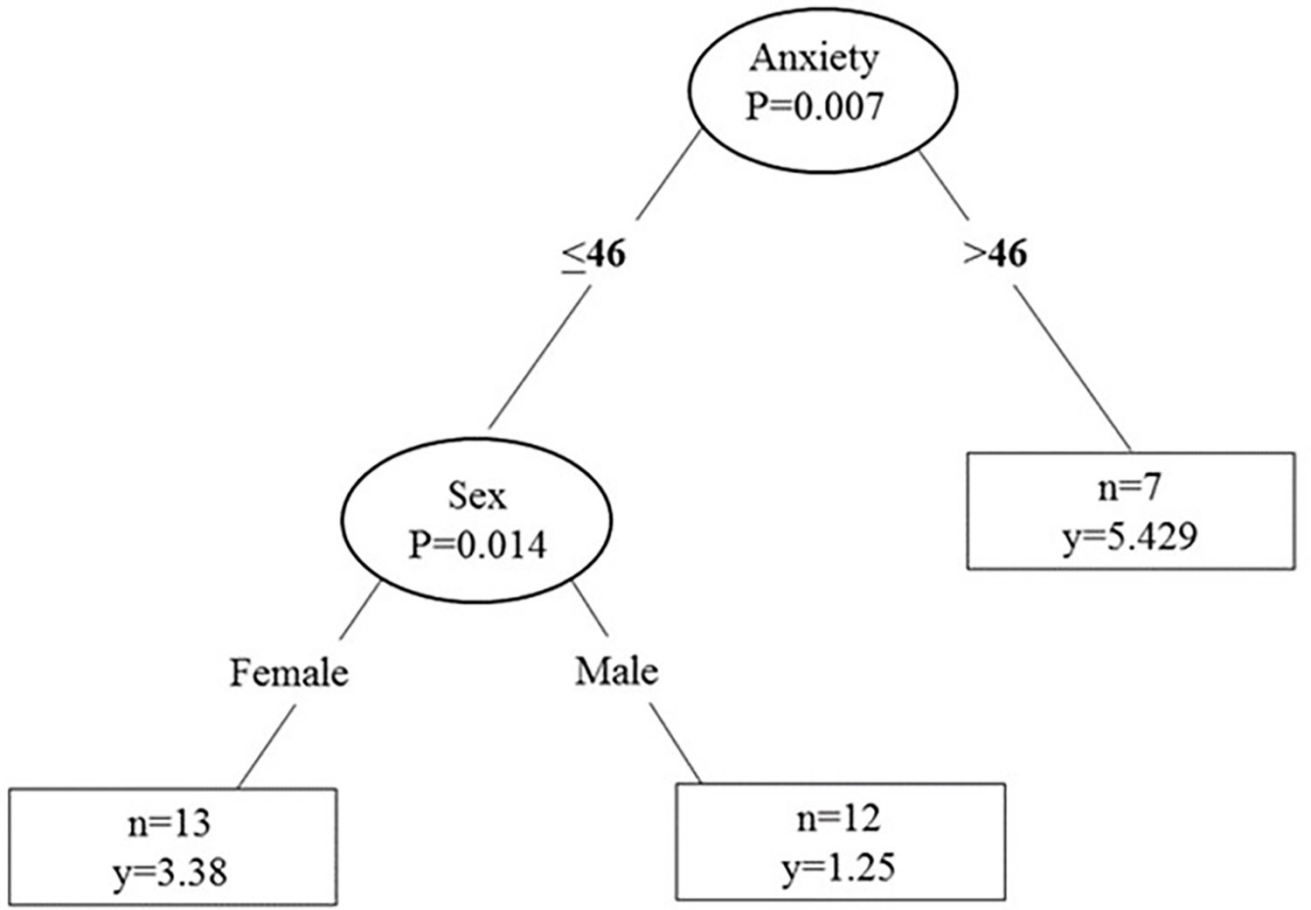
Significant interaction (time by group) for PPTd. The measurement of pain pressure threshold (mean±standard error) on the Deltoid (PPTd) showed that responders (black solid line) demonstrated a significant decrease in PPTd following eccentric exercise. PPT differences between the sessions were 101kPa for responders; 63 kPa for non-responders (brake black line); and -3kPa for the control group who did not perform the exercise (grey solid line).

A mixed model ANOVA (Table 4) revealed that the main effect of time (1^st^ and 2^nd^ session), was significant in each statistical model PPTd, PPTb, and PPTf (p = 0.003, p = 0.0002, and p = 0.0007, respectively). The main effect for group was also identified for the PPTf and PPTb (p = 0.0009 and p = 0.010, respectively). Furthermore, a significant interaction (time by group) was found only for PPTd (p = 0.004). A simple mean analysis, (after Bonferroni correction for 6 comparisons the p-value was set to <0.008) showed that only the responders demonstrated a significant decrease in PPT (responders: M_diff_ = 101.80,t_16 =_ 6.15, CI:(66.73,136.89), p<0.001, *d*z = 0.90; non-responders: M_diff_ = 38.45,t_13 =_ 1.23, CI:(-29.05,105.96), p = 0.24, *d*z = 0.34; Con: M_diff_ = 3.09,t_16 =_ 0.13, CI:(-55.35,49.16), p = 0.90, *d*z = 0.012) (Fig 3).

**Table 4:** PPTs values for responders, non-responders and controls at baseline (1^st^ session) and 24 hours following DOMS induction (2^nd^ session).

	Responders		Non-responders		Controls		Main effect of time	Main effect of group	Main effect Interaction
	1^st^ Session	2^nd^ Session	1^st^ Session	2^nd^ Session	1^st^ Session	2^nd^ Session			
	Mean (SD)	Mean (SD)	Mean (SD)	Mean (SD)	Mean (SD)	Mean (SD)			
**PPTf kPa** (remote area)	361.5	301.0	496.5	420.8	457.4	435.2	*F (1, 41.3) = 13.36, ɳ^2^_p_ = 0.117	*F (2, 29.9) = 9.01, ɳ^2^_p_ = 0.152	F (2, 36.5) = 1.07
	(94.3)	(92.4)	(80.8)	(128.3)	(191.2)	(237.9)			
**PPTb kPa** (local area)	254.1	217.0	355.5	268.4	370.0	345.3	*F (1, 40.9) = 17.36, ɳ^2^_p_ = 0.147	*F (2, 23.5) = 5.96, ɳ^2^_p_ = 0.10	F (2, 31.2) = 1.43
	(77.54)	(71.1)	(72.0)	(108.4)	(199.3)	(185.0)			
**PPTd kPa** (local area)	350.7	246.3	354.8	308.3	442.1	450.5	*F (1, 34.1) = 10.13, ɳ^2^_p_ = 0.091	F (2,30.1) = 1.78,	*F (2,31.1) = 6.65, ɳ^2^_p_ = 0.117
	(106.3)	(121.1)	(106.3)	(130.1)	(266.3)	(255.9)			

PPT, pressure pain threshold; PPTf, pressure pain threshold left flexor; PPTb, pressure pain threshold right biceps; PPTd, pressure pain threshold right deltoid.

*denotes significant main effect p<0.05.

At the remote area (contralateral forearm), we did not find an interaction between time and group for the PPTf. Because this was a secondary exploratory analysis in which we had a major interest in the determining whether DOMS responsivity specifically and individually resulted in change over time, we performed a simple mean analysis and found that only responders demonstrated a significant decrease in PPTf (responders: M_diff_ = 60.47, t_16 =_ 3.28, CI:(21.42,99.51),p = 0.024, *d* = 0.65; non-responders: M_diff_ = 75.73, t_14 =_ 2.58, CI:(12.86,136.61), p = 0.132, *d*z = 0.68; controls: M_diff_ = 22.28, t_18 =_ 0.85, CI:(-32.83,77.39), p = 0.410, *d*z = 0.10).

In order to characterize responders vs. non-responders to DOMS-related MEP we compared the pain sensitivity between the groups at baseline (See Table 4, 1^st^ session) and found that responders demonstrated lower PPTs than non-responders for the PPTf and PPTb (p = 0.0001, d = 1.55 and p = 0.004 d = 1.12, respectively).

For further characterization of responders vs. non-responders, comparisons for the psychological questionnaires’ scores were performed between groups (Table 5). Results show that responders had higher scores for anxiety trait depression and stress symptoms.

**Table 5:** Baseline scores of psychological questionnaires for responders and non-responders to DOMS-related MEP.

Psychological questionnaire	Responders Mean (Q1-Q3)	Non-responders Mean (Q1-Q3)	p-value
**Pain catastrophizing** (PCS)	24.0 (14.0;35.0)	22.0 (16.5;31.0)	0.91
*Trait anxiety (STAI)	46.0 (42.0;51.0)	41.0 (40.5;43.5)	0.03
**State anxiety** (STAI)	44.0 (40.0;46.0)	45.0 (40.5;43.5)	0.43
*Depression (BDI)	10.00 (5.00;13.0)	5.00 (3.0;8.0)	0.03
**Fear of pain** (FPQ)	77.0 (70.5; 94.5)	74.0 (64.0;84.0)	0.61
*Perceived Stress Scale (PSS)	23 (21.5; 25.0)	19.5 (16.8; 21.5)	0.007

PCS, pain catastrophizing scale; SCQ, situational catastrophizing questionnaire; STAI, Spielberger’s State-Trait Anxiety Inventory; BDI, Beck Depression Questionnaire; FPQ, Fear of Pain Questionnaire; PSS, Perceived Stress Scale.

* denotes significant differences between groups p<0.05.

### Prediction of DOMS response

**Fig 4:**
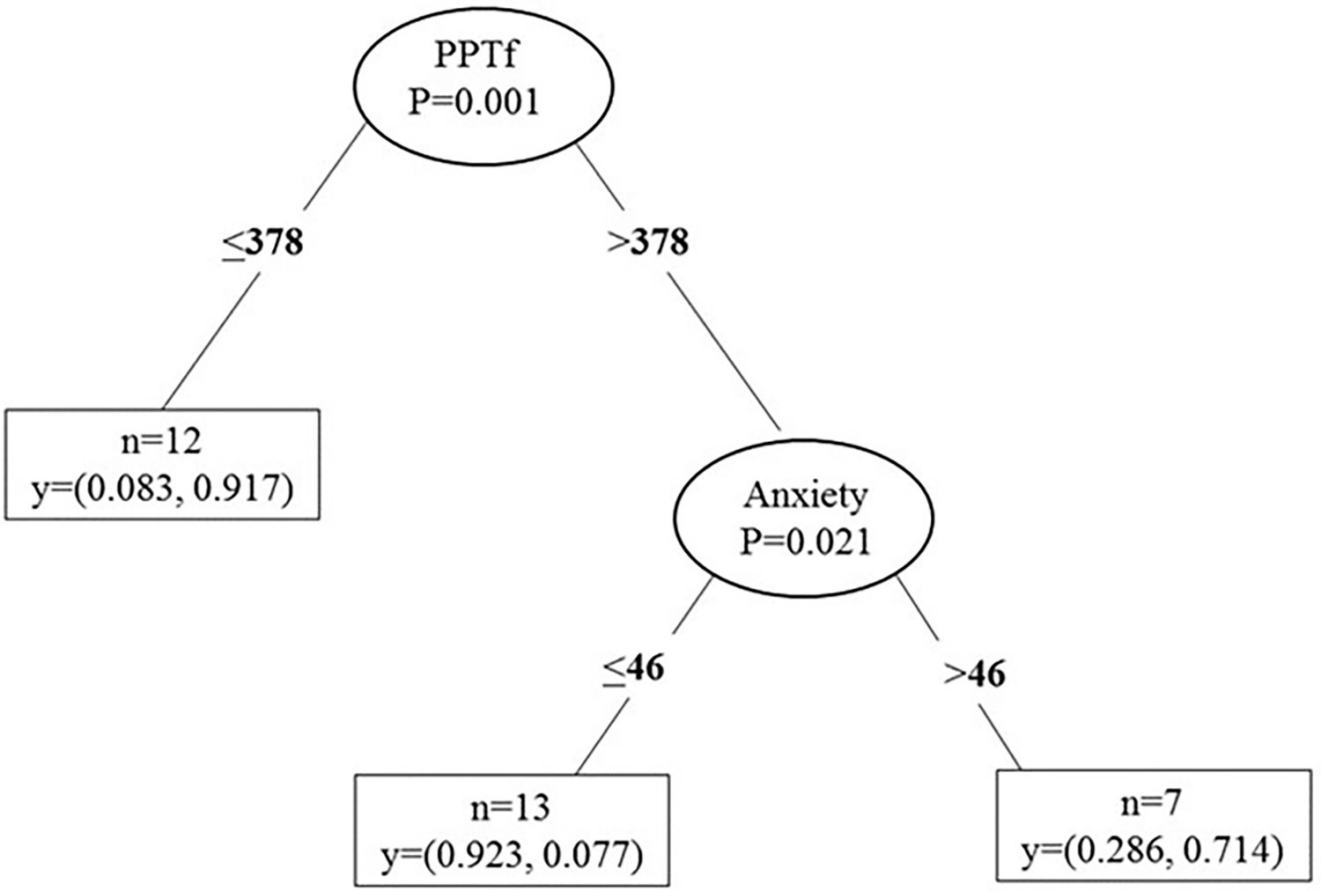
The tree decision analysis to predict DOMS responders. Decision tree analysis set up to predict DOMS responders (VAS≥3) by PPTf (pressure pain threshold in the forearm), below or above 378kPA, and anxiety score below or above 46. The numbers in the circles reflect the level of statistical significance (P); numbers in the rectangular boxes reflect the total number of cases for that outcome (n) and the probability (y) to become a non-responder (first number) or DOMS–responder (second number).

The tree decision analysis (Fig 4) revealed a significant split in the PPTf variable such that participants with a value ≤378kPa at baseline showed a higher probability to become a DOMS responder (this was true for n = 12 participants). Participants with a value >378kPa at baseline for PPTf and an anxiety score >46 also showed a higher probability to become a DOMS responder (this was true for n = 7 participants).

## Discussion

By using a protocol for DOMS induction and focusing on provoked MEP as an outcome measure, we found that among all the pain and psychological measures tested, the level of anxiety trait was the only measure that predicted a higher intensity of MEP, 24 hours following eccentric exercise. In addition, females with mild to moderate levels of trait anxiety were prone to develop greater MEP intensities than males, even after controlling for muscle load during the DOMS induction. However, only 50% of our cohort developed MEP at a mild intensity (i.e. ≥ 3/10 VAS; responders). In order to understand who is at high risk of developing MEP, we investigated the individual pain sensitivity profile and psychological characteristics of these responders. Our novel finding is that systemic muscle pain hypersensitivity (i.e. lower PPTs) and heightened psychological distress (i.e. trait anxiety) predicted the probability to become a responder. The responders also showed a significant increase in muscle pain sensitivity following DOMS induction mainly in the exercised muscle and to some extent in a remote area, suggesting peripheral and central neuroplasticity (i.e., central sensitization), respectively.

Of the many studies that have used DOMS protocols, only a few have referred to non-responders after an exercise protocol for pain induction and reported that ~15–20% of their cohort did not respond with muscle soreness [25, 46, 47]. However, these studies did not specify responders based on a standardized task that elicited MEP and referred only to pain recalled through self-report questionnaires without provocative movement. Assessment of pain in general and not specifically in relation to movement does not evaluate the main clinical characteristic of DOMS. Likewise, questionnaires that are based on individuals’ recall are unable to differentiate pain in relation to movement from other types of pain [7, 48]. In the current study we used mild pain intensity (3 and above on a 0–10 NPS) in response to the MEP test (i.e. lifting a 3kg canister) as the threshold criterion for DOMS responders based on Mankovsky-Arnold et al. [32]. This is an advantage over many other previous studies which did not have such a threshold criterion, meaning that even participants with an incredibly low DOMS intensity were included in their group analysis.

We found that in a healthy population not customized for upper limb eccentric exercise, those who develop MEP are characterized as having basic systemic deep somatic tissue hypersensitivity i.e., lower PPTs in both exercised and remote body muscles. Indeed, PPTs are considered to predominantly reflect the sensitivity of deeper structures [49]. Furthermore, the clinical application of the PPT test has been validated for evaluating pain sensitivity [50, 51] and to identify group differences in pain sensitivity [52, 53]. In the current study, we found that PPT also predicted the probability of MEP occurrence. This finding is in line with the findings of a few previous studies that investigated the predictive ability of PPT in muscular pain [54–56]. Our finding that PPT did not predict DOMS intensity is in line with another study that showed that temporal summation of pain but not PPT predicted DOMS intensity recalled through self-report questionnaires [25]. Taken together, our results emphasize the role of muscular tissue sensitivity in the prediction of the occurrence of MEP following DOMS induction.

The findings of this study have highlighted the significant influence of trait anxiety on the tendency to develop MEP and its intensity. Trait anxiety is a constant future-oriented state characterized by negative affect and anticipation of an unpredictable threatening event. Previous reports suggest that pain-related fear predicts pain intensity [57], physical performance, and interference with life activities following DOMS induction [34, 58]. Furthermore, an interaction between the fear of pain and pain catastrophizing has been shown to predict experimental muscle pain (i.e. intramuscular acidic infusion) intensity and referred pain intensity [56]. Specifically to DOMS, the experience of multisite pain [31] and evoked pain intensity in remote body areas [30] were predicted by fear of pain and pain catastrophizing. Yet in these studies, anxiety trait was not tested as a predictor and the outcome measures were pain in remote areas. In our study, trait anxiety was shown to have a consistent and pronounced correlation to the presence of DOMS-related MEP and its intensity in the exercised muscles. This is in line with previous studies that concluded that not only does trait anxiety have an excitatory effect on pain and vice versa, it also contributes to the intensity of experienced pain [56, 59, 60]. To summarize, our finding that both lower PPTs and high anxiety levels predict MEP is supported by another study that found a cumulative impact of psychological and sensitization risk factors (i.e., high pressure sensitivity measured by PPT) on pain-related outcomes [61].

We found that responders showed significantly decreased PPTs (i.e., mechanical allodynia) in the exercised muscles. This is a result of peripheral sensitization of intramuscular nociceptors from algogenic substances released subsequent to muscle damage [15, 16, 62]. In addition, the inflammation that occurs in the region of damage may turn on silent nociceptive neurons that become activated in response to normally innocuous movement [63]; a mechanism that may explain MEP. Moreover, the responders also demonstrated some reduction in PPT in the non-exercised muscle, suggesting the development of central sensitization [14, 47, 64–67]. Indeed, MEP is a clinical manifestation of mechanical allodynia that may also arise from central sensitization that causes altered processing of mechanoreceptor afferent activity [11]. Thus, the coupling between MEP and decreased PPTs in remote body areas suggests that they are phenotypes of central sensitization [13]. The development of central sensitization following DOMS induction is also supported by previous reports of increased temporal summation of pain stimuli. [64, 67, 68], increased frequency of referred pain, and enlarged pain areas [47]. Thus, we suggest that both peripheral and central mechanisms are at the base of above mild intensity MEP. Moreover, we carefully suggest that the MEP test may serve as a diagnostic tool of pain hypersensitivity due to central neural plasticity [14].

This study has several limitations that need be taken into consideration for future studies. We cannot rule out the possibility that DOMS occurred in some participants for whom the MEP task did not evoke pain of 3 or greater. In addition, DOMS is a progressive process which reaches a peak 24–72 hours after exercise regimen. In the current study we measured pain sensitivity only after 24 hours. It is possible that later measurements along the time course of DOMS development will shed more light on the pain neuroplasticity progress. Furthermore, the current findings are limited by the moderate sample size, thus validation of the present findings with larger sample sizes are needed. Finally, DOMS is a type of acute muscle pain that is evoked by movement and therefore is a specific clinical condition that exemplifies the reciprocal relationships of pain and movement. Thus, the conclusion of this study regarding the mechanisms associated with MEP cannot be separated from those of DOMS.

## Conclusions

This is the first study to characterize DOMS responders and non-responders that were classified based on a provoked MEP test and to predict those who will develop DOMS following eccentric exercise. Our findings show that high anxiety trait increases the probability to develop DOMS-related MEP and together with greater systemic muscle pain sensitivity it increases MEP intensity. Furthermore, once DOMS is established at a higher than mild intensity level of MEP, it is accompanied by systemic muscle hypersensitivity, suggesting peripheral and central neuroplasticity.

This knowledge is valuable in translating science into clinical musculoskeletal pain management. DOMS is considered an experimental model for clinical musculoskeletal pain and the same factors that shape responses to experimental pain stimuli also contribute to the experience of clinical pain. Therefore, by applying the DOMS as an acute muscle pain model in a clinical trial, putative risk factors can be assessed in order to identify those who are vulnerable to DOMS-related MEP.
